# Nutrient Intakes Status and Physical Inactivity among Overweight and Obese School Children in Kota Bharu, Kelantan, Malaysia

**Published:** 2018-08

**Authors:** Wan Putri Elena WAN DALI, Hamid Jan JAN MOHAMED, Hafzan YUSOFF

**Affiliations:** Nutrition and Dietetics Programme, School of Health Sciences, Universiti Sains Malaysia, Kota Bharu, Kelantan, Malaysia

**Keywords:** Overweight, Obesity, School children, Nutrient intakes, Physical activity

## Abstract

**Background:**

The study examined the baseline findings of a controlled intervention study comprising anthropometric measurements, nutrient intakes, and physical activity among overweight or obese children in Kota Bharu, Kelantan, Malaysia.

**Methods:**

The study was completed in 2016 and the baseline data were gathered from four groups in a school-based randomized community trial among Year Five students from primary schools in Kota Bharu, Kelantan, Malaysia. Participants completed anthropometry assessment, three-day dietary record, and Physical Activity Questionnaire for Older Children (PAQ-C).

**Results:**

The prevalence of obesity was higher among the boys (52.5%). Mean energy intake was significantly higher among boys as compared to the girls (*P*=0.003). Twenty-five percent of the participants had exceeded the recommended nutrient intakes (RNI) of energy recommended. The calcium, thiamine, riboflavin, and niacin were also significantly higher among boys as compared to the girls (*P*<0.05). Boys also exhibited a significantly higher score on performance of physical activity (mean=2.68; SD=0.60) as compared to the girls (mean=2.38; SD=0.51) however it is still in the category of moderately active. Approximately 14.4% of children had a very low physical activity level.

**Conclusion:**

Overweight and obese boys had higher energy and fat intakes but were more physically active as compared to the girls. These findings might be useful in planning appropriate intervention strategies to be designed and delivered especially for this cohort.

## Introduction

Poor eating habits such as skipping meal (particularly breakfast), failure to achieve fruits, and vegetable intake recommendation and high frequency of fast food consumption are major public health concerns among children (1,2). Eating breakfast is crucial for children’s health and development. Unfortunately, in Kuala Lumpur, the most frequently missed meal was breakfast (12.6%), followed by lunch, (6.7%) and dinner (4.4%) (1). Consumption of the Western fast foods was also very popular among the children where 60% to 70% of them consumed fast food in the week prior to the study conducted by Moy and researchers (1).

Kelantan cuisines contain a lot of sugar and coconut milk, making most dishes in Kelantan to be sweet and creamy (3). The sugary and creamy cuisines are often associated with long-term chronic diseases. Similar condition was also reported in Malacca, Malaysia which showed that the high amount of coconut milk consumption had resulted in young patients having early heart disease, hypertension and kidney disease (4). However, the evidence-based Malaysian food towards long-term chronic diseases still remains unclear. Low fruits and vegetable consumption by the children are another pressing matter. Even among Malaysian adults, the consumption of fruits is still low and the beneficial food is not included in the top ten daily consumed foods (5). Among 34 countries across five WHO Regions that participated in the Global School-based Student Health Survey (GSHS) (6), only 23.8% of boys and 15.4% of girls aged 13 to 15 yr were classified as physically active which majority of the children did not meet the physical activity recommendations (7). This GSHS study was conducted between year 2003 to 2007 and involved a total of 72845 children from American regions (Argentina, Cayman Islands, Chile, Colombia, Ecuador, Guyana, Saint Lucia, Saint Vincent and the Grenadines, Trinidad and Tobago, Uruguay and Venezuela), African regions (Botswana, Ghana, Kenya, Mauritius, Namibia, Senegal, Seychelles, Uganda, UR Tanzania, Zambia, and Zimbabwe), Eastern Mediterranean regions (Djibouti, Egypt, Jordan, Libyan Arab Jamahiriya, Morocco, Oman, and United Arab Emirates), South-East Asia regions (India, Indonesia and Myanmar) and Western Pacific regions (China and Philippines). It is worrisome as the children will become more sedentary as they get older (8). Unhealthy dietary practices with lack of physical activity may have been contributed to the children to become at risk of being overweight and obese (9). In addition, obese children tend to become obese adolescents and obese adults at later age. Over-weight and obesity determinants among children are critical issues that need to be investigated in order to prevent undesirable weight-related health conditions in individuals and in the society.

Nutrition education has been recognized as a crucial component in any programs and interventions related to health promotion and disease prevention (10). Nutrition education delivered to children population has not only been shown to improve the nutrition knowledge, attitude and practice (10) but also in obesity-associated behaviors, including physical activity, dietary behaviors and screen time (11). Implementation of interventions for overweight and obese children population are also effective in reducing weight gain and reduce the prevalence of obesity (12).

The objective of this study was to present the baseline results of a controlled intervention study assessing the anthropometric measurements, dietary intakes, and physical activities among over-weight or obese school children in Kota Bharu, Kelantan, Malaysia.

## Materials and Methods

### Study design

This investigation was based on four-group school-based randomized controlled trial actually used for the intervention study later. Twenty out of 96 schools in Kota Bharu were randomly approached with 2986 total number of student. However, 6 schools were excluded either due to less cooperation obtained from the school’s administrator or having no overweight and obese category of children in those schools. There were only 139 children who met the inclusion and exclusion criteria and voluntarily consented to take part in this study.

### Participants

Participating school children fulfilled our inclusion criteria were: from year five from primary schools, being overweight or obese, generally healthy without any chronic diseases, and able to complete the questionnaires given.

All parents were provided written consent form and children were also provided informed assent form. All the children and their parents/guardians were informed about the objectives, procedures, potential risks, and benefits of the study.

### Sample size calculation

Data collection was conducted during the period of Sep 2015 until Nov 2016. Sample size calculation was determined using Daniel’s formula (13):

$$n\Delta =z^{2}\left\{\text{x}\_p\right\}\;\left(1-p\right)$$

Where *n*= required sample size; *p*= percentage for school children in Kelantan who were at risk of overweight (13.1%) (14); Δ= detectable difference which was set at 6.55% (9); z = confidence interval of 95%, which is 1.96. After considering a 20% dropout, the minimum number of participants required was 122.

### Outcomes

#### Personal details

Such as sex, residential zone, monthly household income, parent’s occupation, information about obese family members, and frequency of taking breakfast were obtained using a standard questionnaire.

#### Anthropometric measurements

Weight, height, and waist and hip circumferences, and body fat mass were taken in duplicate by a trained researcher. The body weight was determined to the nearest 0.5 kg on an electronic digital scale (TANITA Body Composition Analyzer SC-330) and height was measured to the nearest 0.1 cm using a body meter (SECA 206). For the waist circumference, the inferior margin (lowest point) of the last rib and the crest of the ilium (top of the hip bone) were identified. All of these measures were collected by a single evaluator, who constantly utilized the same routinely calibrated equipment’s. Body mass index (BMI) was derived using the following equation: weight in kilogram divided by height in meter square; BMI = weight (kg)/height (m^2^). Subsequently, BMI results were categorized according to the WHO Reference BMI-for-age growth charts (15). For children, overweight is defined as having a BMI above >+1SD whereas above >+2SD is qualified as obesity.

### 3-day diet record form

Information on eating habits was obtained using a three-day diet record (consisting two weekdays and one day on the weekend). The children were asked to record all details including mealtime, type of foods and drinks, cooking methods, estimated portion size with local household measurement of foods and beverages and dining place starting from the first day of the meeting. Then, the list of foods and beverages consumed in three days were added in the Nutritionist Pro Software (Axxya Systems, USA). The serving size per day of each food and beverage item were also added. Mean energy and nutrient intakes (carbohydrate, protein, fat, calcium, iron, thiamin, riboflavin, niacin, and vitamin C) for the three days were calculated by this software. Under-reporting and over-reporting of energy intake were examined by calculating the ratio between reported energy intake (EI) and basal metabolic rate (BMR). The BMR was calculated using the WHO equation for children (16). EI/BMR <0.76, 0.76–1.24 and >1.24 were interpreted as under-reporting, acceptable and over-reporting of EI respectively (17).

#### Physical activity questionnaire for older children (PAQ-C)

Physical activity assessment was measured using Physical Activity Questionnaire for Older Children (PAQ-C) completed by the children. PAQC was self-administered with seven-day recall instrument that is suitable for children aged 8 to 14 yr old (18). It provided a summary of physical activity scores derived from nine items; each score is based on a five-point scale. This instrument has been widely tested and validated (18). The following values were adopted for the analysis of PAQ-C data: item 1 (“no” activity being a 1, “7 times or more” being a 5); items 2 to 8 (the lowest activity response being a 1 and the highest activity response being a 5) and item 9 (“none” being a 1, “very often” being a 5). Item 1 provides a checklist which composed of over twenty physical activities. The students were asked on how many times they carry out for each type of physical activity in the past 7 days. Item 2 to 7 describe their activity level in different school settings [physical education (PE), recess, lunch, right after school, evening and weekends]. While the item 8 (describe you best) requires the students to summarize their general activity levels based on the five different statements. Item 9 refer to the mean total of all days of the week.

Then, all nine items were summed and divided by nine and were resulted to the final PAQ-C activity summary score. Then, the scores were classified into three categories; 1 to 2.33 as low, 2.34 to 3.66 as moderate and 3.67 to 5.00 as high (19).

### Ethical approval

Initially, the present study was approved by the Universiti Sains Malaysia Human Research Ethics Committee (USM/JEPeM/14110478), Ministry of Education (MoE), Kelantan Education Department and all the school’s administration department of primary schools in Kota Bharu.

### Statistical analysis

Data were compiled and analyzed using IBM SPSS version 20.0 (Chicago, IL, USA). Normal distribution of data was checked using Kolmogorov-Smirnov normality test. Independent t-test was used to compare mean score and sex while the Chi-square test was used to test the association between two categorical variables. Means and standard deviations were calculated for each variable and the differences at *P*<0.05 were considered as significant.

## Results

Table 1 summarizes the characteristics of the participants. Among the entire participants of 139 children, (i) 81.3% were obese, (ii) 61.9% were boys, (iii) all of them were Malays and (iv) more than half of the participants were from the suburban areas (53.2%).

**Table 1:** General characteristics of the participants (n=139)

***Socio-demographic variables***	***Boys, n=86 (%)***	***Girls, n=53 (%)***	***Total, N=139, (%)***	***x^2^ (df)***	P-value*
**Residential zone**				4.098 (1)	0.143
Urban	46 (33.1)	19 (13.7)	65 (46.8)		
Sub-urban	40 (28.8)	34 (24.5)	74 (53.2)		
**Occupation**					
**Father**					
Not working	11 (7.9)	13 (9.35)	24 (17.3)		
Government sector	31 (22.3)	13 (9.35)	44 (31.7)		
Private sector	18 (12.95)	13 (9.35)	31 (22.3)		
Own business	26 (18.7)	14 (10.1)	40 (28.8)		
**Mother**					
Not working	31 (22.3)	23 (16.5)	54 (38.8)		
Government sector	32 (23.0)	17 (12.2)	49 (35.5)		
Private sector	2 (1.4)	4 (2.9)	6 (4.3)		
Own business	21 (15.1)	9 (6.5)	30 (21.6)		
**Household income**				2.331 (1)	0.127
Below MYR3000	56 (40.3)	41 (29.5)	97 (70.5)		
Above MYR 3000	30 (21.6)	12 (8.6)	42 (29.5)		
**BMI status**				2.304 (1)	0.129
Overweight	11 (9.4)	12 (8.6)	25 (18.0)		
Obese	75 (52.5)	41 (29.5)	114 (82.0)		
**Family members who are over-weight/obese**				2.409 (1)	0.121
Yes	54 (38.8)	40 (28.8)	94 (67.6)		
No	32 (23.0)	13 (9.4)	45 (32.4)		
**Frequency of breakfast**				0.509 (2)	0.775
Every day	43 (30.9)	25 (18.0)	68 (48.9)		
2 – 5 days per week	26 (18.7)	19 (13.7)	45 (32.4)		
Almost never	17 (12.2)	9 (6.5)	26 (18.7)		

* Chi-square test

As for the family economic status, the highest percentage of the family income was shown in the range between RM1001 – RM3000; in majority, both parents worked either in private or government sectors in Kota Bharu, Kelantan. Almost half proportion of respondents skipped their breakfast daily 48.9% (n=68).

**Fig. 1:**
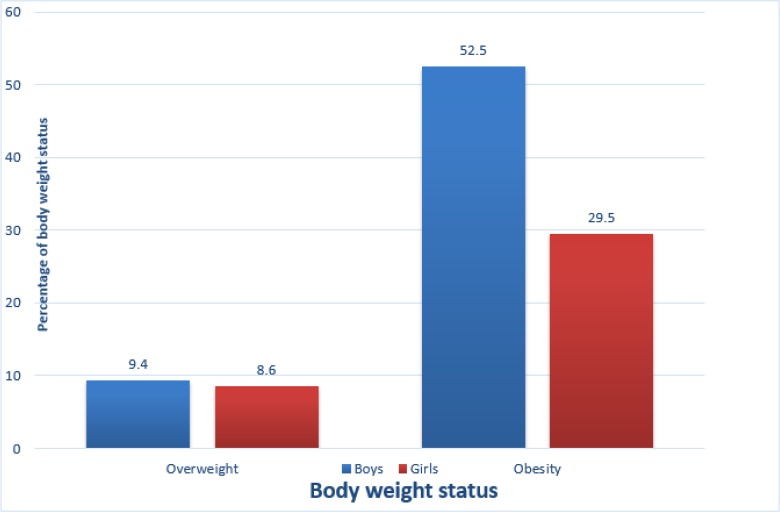
Association between gender and BMI status (χ^2^=0.873; *P*=0.350)

Overall, the mean height, weight, BMI and fat mass were 144.61 cm, 53.94 kg, 25.96 kg/m^2^ and 22.37 kg respectively (Table 2). The boys had significantly higher measurement of waist circumference compared to the girls (*P*=0.034). Based on the cut-offs set by WHO (15), 18.7% of the participants were overweight, and 81.3% were obese in this sample. When analyzed by sex, 16.3% of the boys and 22.6% of the girls were overweight while 83.7% of the boys and 77.4% of the girls were obese (Fig. 1).

**Table 2:** Body composition of the participants (n=139)

***Variables***	***All, Mean (SD)***	***Boys, (n=86) Mean (SD)***	***Girls, (n=53) Mean (SD)***	***P-value***
Height (cm)	144.61 (6.34)	144.01 (6.10)	145.59 (6.65)	0.153
Weight (kg)	53.94 (9.11)	54.93 (9.45)	56.09 (9.07)	0.474
BMI	25.96 (3.64)	26.42 (3.80)	26.37 (3.32)	0.946
Waist circumference (cm)*	83.46 (7.33)	85.6 (8.34)	82.75 (6.35)	0.034
Hip circumference (cm)	93.16 (6.97)	93.33 (7.15)	94.50 (6.78)	0.338
Fat mass (kg)	22.37 (8.63)	23.28 (9.33)	22.76 (8.16)	0.729

* *P*-value <0.05; Independent *t*-test

Nonetheless, no significant association was found between sex and BMI categories (χ^2^=0.873; *P*=0.350). The participants were asked to complete the three-day diet record.

However, 17.3% (n=24) of children did not complete the three-day diet record and therefore has been excluded in the analysis. Energy misreporting was prevalent in 55 of 115 children (47.8%); nine children under-reported, whereas 46 children over-reported. The actual food intakes consumed by under-reporters and over-reporters were declared by their parents and teachers in school. The mean daily intakes of macronutrients and micronutrients are presented in Table 3. The mean energy intake was significantly higher among boys compared to girls (*P*=0.003). This overweight and obese group [17.4% boys (n=15) and 26.4% girls (n=14)] had exceeded the RNI for energy recommended for their age group. Regarding the National Academy of Sciences, (20), the acceptable macronutrient distribution ranges (AMDR) for children aged 9 to 13 yr are 45%–65% for carbohydrates, 10%–30% for protein and 25%–35% for fat.

**Table 3:** Energy and nutrient intakes according to sex (n=115)

***Nutrient (unit)***	***Boys (n = 68) mean (SD)***	***RNI/AMDR value for boys***	***Girls (n=47) mean (SD)***	***RNI/AMDR value for girls***	***Macronutrient distribution (%)***	***P-value^a^***
Energy (kcal)	1886.83 (502.44)	2180	1671.58 (423.66)	1990	-	0.018
More than RNI, n (%)	15 (17.4)		14 (26.4)		-	
Carbohydrate (g)	234.77 (69.61)	45–65%*	208.73 (54.94)	45–65%*	49.8; 49.9	0.034
Protein (g)	71.54 (24.34)	10–30%*	67.20 (18.96)	10–30%*	16.1; 15.2	0.307
Fat (g)	74.41 (26.24)	25–35%*	63.60 (24.25)	25–35%*	35.5; 34.2	0.027
Calcium (mg)	507.28 (218.58)	1000	422.21 (231.93)	1000	-	0.048
Iron (mg)	21.56 (9.39)	15	19.10 (8.84)	14	-	0.160
Thiamine (mg)	1.11 (0.41)	1.2	0.91 (0.44)	1.1	-	0.014
Riboflavin (mg)	1.87 (0.91)	1.3	1.47 (0.83)	1.0	-	0.017
Niacin (mg)	17.55 (6.30)	16	14.75 (5.62)	16	-	0.016
Vitamin C (mg)	57.16 (49.45)	65	59.55 (66.24)	65	-	0.825

a Independent *t*-test; RNI = Recommended Nutrient Intakes;

* AMDR=Acceptable macronutrient distribution ranges

The protein and carbohydrate intakes were in the acceptable range but fat intakes among boys slightly exceeded the recommended range at 35.5%. Similarly, calcium, thiamine, riboflavin and niacin intakes were also significantly higher among boys compared to the girls (*P*<0.05). Table 4 shows the mean value of physical activity by comparison of sexes. The boys were significantly more physically active than the girls as exemplified by the PAQ-C mean scores (*P*=0.003).

**Table 4:** Summary score for the PAQ-C according to sex (n=139)

***Variables***	***All (n=139)***		***Boys (n=86)***		***Girls (n=53)***		***P-value^a^***
	***Mean***	***SD***	***Mean***	***SD***	***Mean***	***SD***	
Checklist	1.81	0.48	1.84	0.51	1.75	0.42	0.276
PE class	3.74	1.05	3.92	1.10	3.45	0.91	0.011
Recess	2.01	1.10	2.16	1.23	1.75	0.81	0.019
Lunch	1.93	1.08	2.03	1.14	1.75	0.96	0.138
After school	2.84	1.24	2.98	1.26	2.62	1.16	0.101
Evenings	2.90	1.13	3.10	1.15	2.57	1.03	0.006
Weekend	3.04	1.14	3.14	1.18	2.87	1.06	0.173
Describes best	2.02	0.98	2.00	1.06	2.06	0.95	0.743
Week summary	2.80	0.95	2.94	0.97	2.56	0.88	0.021
PAQ-C*	2.56	0.59	2.68	0.60	2.38	0.51	0.003

Each item is scored on a five-point scale with higher values indicating higher activity,

PAQ-C* is average of nine items;

a Independent *t*-test

Specifically, the boys were also significantly active during PE classes (boys=3.92 ± 1.10; girls=3.45 ± 0.91; *P*=0.011), recesses (boys=2.16 ± 1.10; girls=1.75 ± 0.91 *P*=0.019), evenings (boys=3.10 ± 1.15; girls=2.57 ± 1.03; *P*=0.006) and in week summary (boys=2.94 ± 0.97; girls=2.56 ± 0.88; *P*=0.021) compared to the girls. However, the mean of PAQ-C for both sexes was considered moderately active (mean=2.56).

## Discussion

The data provides information on the prevalence of overweight issue and obesity, dietary intakes as well as the physical activity levels of overweight and obese school children in Kota Bharu, Kelantan. The percentage of obese children was higher than overweight children and the number of boys was higher than girls in the obese group.

Similar findings on the prevalence of obesity among boys (66.7%) as compared to girls (33.3%) were reported (21). Among 278 Chinese school children in Kota Bharu, only four children were overweight, while 65 children were obese; 44 of the obese children were boys (22). Higher reported prevalence of obesity in boys most probably related to the differences in body composition, patterns of weight gain, hormone biology and to the certain social, ethnic, genetic and environmental factors (23). In our sub-analysis, high prevalence of obese boys was demonstrated by those residing in urban area (28.8%). Parents who were working in the urban area have long working hours and therefore are unable to prepare home-made meals for their child, reflecting in higher tendency of having meals with high energy and saturated fat at the hawker stalls and fast-food restaurants (24). The girls were more concerned about their weight and body shape than boys (25). Furthermore, parents are less likely to encourage their son to lose weight, perhaps due to their perception that the body size will result in more muscular and ideal male body shape (26).

Overweight and obese children in the present study were more likely to skip breakfast compared to children who have normal BMI in other studies (1, 22). Skipping breakfast was associated with increased prevalence of overweight issue and obesity (27, 28). Individuals who skipped breakfast tended to be hungrier and may eat larger portions during the next meal (29). Apart from no appetite in the morning, other reasons for skipping breakfast included no breakfast was prepared, no time for breakfast and lack of awareness on the importance of breakfast (22). Having overweight family members’ is associated with increase in the children’s BMI. This finding is parallel with another study that showed the prevalence of overweight issue in children increased proportionally with guardians’ BMI status (24). Additionally, the children are at higher risk and chance to be obese if both parents were obese (30).

Assessment of diets was met with several difficulties including the tendency to gather under- reported energy intake shown to be more common in overweight and obese children (31). In total, reported energy intake during baseline was lower than expected and this is in line with previous studies conducted in Malaysia (22, 32). This may be attributed to social pressures including homework or challenges in completing the three-day diet record as requested (33). Notably, few children exceeded the energy and fat intake. Causes of increased energy and fat intakes include larger portion sizes, eating in restaurants and away from home, eating late at night, instant-availability of energy-dense foods and fast foods and frequent snacking as well (34). With high living costs and hectic lifestyle especially in urban city, there is a tendency for peoples choosing cheaper-priced food over expensive one (35).

In terms of physical activity outcomes, the boys were observed to be more significantly active than girls especially during PE classes, recesses, evenings and total physical activities. This finding is consistent with the results of another study (36). One of the major reasons is that girls have earlier rapid decline in physical activity than boys. This gender difference was started from as early as 10 yr old (37), whereas other study claimed the ages to be from 12 to 16 and another study observed it in the age of 11 yr old (36, 38). Nevertheless, the highest decline of physical activity took place during adolescence, from ages 15 to 18 (36, 39). Furthermore, children tend to become less active as they get older with more of their time spent sitting in front of the computer, watching television or playing a wide range of inactive video games. Reducing these sedentary activities to less than two hours per day is important to increase child’s physical activity and health (40). Notably, the total physical activities for both overweight and obese boys and girls in this latest finding were considered as moderately active. Thus, implementation of the exercise intervention is urgently needed.

This study relied on the self-reported measure for physical activity outcome and self-records for food and beverage intakes. This information were highly dependent on the participants’ memory, honesty and trustworthiness in answering the questions. The under-reporting and over-reporting of individual dietary data were unavoidable. However, this bias was mitigated by using the deep conversation technique such as probing for details of foods consumed, as well as using additional pictures and food models.

## Conclusion

The prevalence of obesity was quite alarming among school children aged 11 yr in Kota Bharu, Kelantan, Malaysia. Overweight and obese children were more likely to skip breakfast and exceed the appropriate energy and fat consumption levels especially boys. However, boys generated higher level of physical activity compared to the girls. Still, the total score of physical activity level of the children in this overweight and obese group was also considered as moderately active. Thus, appropriate action needs to be taken to address all these problems in order to build a strong and healthy generation in the future.

## Ethical considerations

Ethical issues (Including plagiarism, informed consent, misconduct, data fabrication and/or falsification, double publication and/or submission, redundancy, etc.) have been completely observed by the authors.
